# Pediatric Obesity—A Potential Risk Factor for Systemic Inflammatory Syndrome Associated to COVID-19, a Case Report

**DOI:** 10.3389/fped.2021.681626

**Published:** 2021-05-28

**Authors:** Cristina Oana Mărginean, Lorena Elena Meliţ, Maria Oana Săsăran

**Affiliations:** ^1^Department of Pediatrics I, George Emil Palade University of Medicine, Pharmacy, Science, and Technology of Târgu Mureş, Târgu Mureş, Romania; ^2^Department of Pediatrics III, George Emil Palade University of Medicine, Pharmacy, Science, and Technology of Târgu Mureş, Târgu Mureş, Romania

**Keywords:** obesity, children, pediatric inflammatory multisystem syndrome, COVID-19, subclinical inflammatory status

## Abstract

The well-documented systemic inflammation associated to pediatric obesity might act as an augmenting factor for other inflammatory conditions, such as pediatric inflammatory multisystem syndrome (PIMS) associated to COVID-19. We report the case of 9-year-old boy admitted in our clinic for fever, anorexia, and fatigability. The clinical exam revealed influenced general status, palpebral edema, non-exudative conjunctivitis, and abdominal tenderness. The patient weighed 45 kg. The laboratory tests at the time of admission pointed out anemia, lymphopenia; elevated inflammatory biomarkers, NT-proBNP, D-dimers, and troponin; high liver enzymes and lactate dehydrogenase levels, as well as hypoalbuminemia. The patient tested positive for both RT-PCR and serology for SARS-CoV-2 infection. We initiated intravenous immunoglobulin and methylprednisolone, associated with empirical antibiotic, anticoagulation therapy, and symptomatic treatment. The patient was discharged on the 7th day of admission with the recommendation to continue enoxaparin and methylprednisolone at home tapering the dose for the next week. The subclinical inflammatory status associated to obesity might serve as an unfortunate trigger factor for the development of COVID-19 severe forms in children. Therefore, clinicians should be aware that children with obesity and COVID-19 represent a peculiar group that should be closely monitored and thoroughly assessed in order to preempt life-threatening complications, such as PIMS.

## Introduction

The era of pediatric obesity has recently become a worldwide public health problem, being well-known that it represents a multisystem disease with a complex etiology triggered by environmental, biological and societal factors (1). Nevertheless, genetic predisposition might play a key role in the development of severe obesity (1, 2). According to the alarming reports of World Health Organization from 2018, ~40 million children aged under 5 years suffer from overweight or obesity (3). It was hypothesized that adipocytes have the ability to activate immune cells and to further passively participate to the inflammatory processed within different specific tissues (4). Furthermore, the involvement of adipose tissue in the development of systemic inflammatory status is sustained by the two major functions of adipocytes: synthesis and secretion of certain products, among which proinflammatory cytokines and chemokines, defined as chemoattractant and activators of certain immune cells, among which monocytes and polymorphonuclear cells (5). In addition, adipose tissue might be considered to have a dichotomous role acting as both a source of inflammation and a target of inflammatory processes (6). The subclinical inflammatory status associated to obesity can be detected in the peripheral blood of otherwise healthy overweight or obese individuals independently of the age (7, 8). Taking into account all these aforementioned statements, we might hypothesize that an additional inflammation of infectious or non-infectious cause might result in a life-threatening condition in individuals with obesity that already express a subclinical inflammation.

Coronavirus disease 2019 (COVID-19) is a recent multisystem disorder cause by the severe acute respiratory syndrome-2 (SARS-CoV-2), which emerged in December 2019 in China and rapidly become a global burden being declared as pandemic on March 11, 2020 by the World Health Organization (WHO) (9). It was initially reported that only a small proportion of children are affected by COVID-19 and those who develop the disease usually display asymptomatic or milder forms (10). Nevertheless, several countries noticed that small ages might also be predisposed to develop severe forms defined as a severe multisystem inflammatory syndrome similar to Kawasaki disease and Toxic shock syndrome (11, 12). Taking into account the potentially fatal evolution of children who develop this syndrome, complex combined diagnostic criteria were postulated in order to increase clinician's awareness and for an early diagnosis by several associations, such as The Royal College of Pediatrics and Child Health, the WHO, or Centers for Disease Control and Prevention (12–14). Therefore, these experts identified the following criteria as the most common for this multisystem inflammatory status associated to COVID-19 in children with evidence of COVID-19 (real-time polymerase chain reaction—RT-PCR, antigen test, or serology positive) an no other proven cause of inflammation: fever, hypotension or shock, coagulopathy, cardiac, or other organ involvement (renal, respiratory, dermatological, neurological), conjunctivitis, acute gastrointestinal disorders, and elevated inflammatory markers (12–14). The prevention of this pediatric inflammatory multisystem syndrome has become a main focus of health care providers worldwide, and obesity has been identified as a crucial risk factor for severe forms of COVID-19 even in children (15).

The aim of this case report is to underline once more that we currently live in the era of two pandemics: childhood obesity and COVID-19, and their association might be fatal.

*The informed consent was obtained from the patient's mother prior to the publication of this case report*.

## Case Report

### Presenting concerns

We report the case of a 9-year-old boy admitted in our clinic for fever (maximum 39°C), loss of appetite, fatigability with the onset ~6 days before the admission. The anamnesis revealed that the mother was diagnosed with COVID-19 ~1 month ago, but the patient developed no symptoms at that time. We found no previous history of any non-communicable diseases in our patient.

### Clinical Findings

The clinical exam at the time of admission revealed influenced general status, palpebral edema, non-exudative conjunctivitis, and abdominal tenderness. The patient weighed ~45 kg, with a body mass index of 23 (height 140 cm), *Z*-Score 1.6 and percentile 95% being classified as obese.

### Diagnostic Focus and Assessment

The laboratory tests at the time of admission pointed out anemia (Hemoglobin 9.6 g/dL), lymphopenia (1,420/μL); elevated inflammatory biomarkers (C-reactive protein >160 mg/L, erythrocyte sedimentation rate—ESR 50 mm/h, ferritin 487 ng/mL, fibrinogen 615 mg/dL), NT-proBNP (1,899 pg/mL), D-dimers (542 ng/mL), and troponin (21.7 ng/L); higher liver enzymes (aspartate aminotransferase—AST 82 U/L, alanine aminotransferase—ALT 58 U/L) and lactate dehydrogenase levels (377 U/L), hypoalbuminemia (3.49 g/dL). We found no other changes in the coagulation parameters. The thoracic radiography, abdominal ultrasound and echocardiography revealed no abnormalities. The patient tested positive for RT-PCR SARS-CoV-2 infection, and thus we established the diagnosis of pediatric inflammatory multisystem syndrome associated to COVID-19 (PIMS). We found no microbiological evidence of other bacterial or viral infection. The serological test for SARS-CoV-2 infection was positive.

### Therapeutic Focus and Assessment

We initiated intravenous immunoglobulin in a dose of 0.5 g/kg/day for 5 days and methylprednisolone 2 mg/kg/day for 7 days, associated with empirical antibiotic (Ceftriaxone), anticoagulation therapy (enoxaparin) 40 mg twice a day, and symptomatic treatment. The evolution was mildly favorable with the normalization of most of the laboratory parameters after ~7 days, except of ferritin which persisted elevated (613 ng/ml). The RT-PCR test for SARS-CoV-2 was negative in the 6th day of admission. The patient was discharged on the 7th day of admission with the recommendation to continue the methylprednisolone at home in a progressive decreasing dose and enoxaparin 40 mg once a day for the next week.

### Follow-Up and Outcome

The patient expressed a good tolerability and adherence to all administered drugs without any adverse or unanticipated events. The laboratory tests after ~7 days from discharge were within normal limits with only a mildly elevated level of ALT (73.6 U/L) and ferritin (104.6 ng/mL). The follow-up echocardiography remained normal.

## Discussions

PIMS is a rare condition, but extremely severe condition in children and it has been proved to occur up to 4 weeks after the onset of COVID-19 even in initially asymptomatic patients (16). Similarly, our patient developed no symptoms for ~3 weeks after the diagnosis of COVID-19 in his mother. A previous report from July 2020 identified 570 patients with PIMS from March 2 to July 18 2020 in the USA, out of which 203 presented with shock, cardiac impairment, abdominal pain, and severely increased inflammatory biomarkers, while the remaining 367 presented similar manifestations to acute COVID-19 with a less severe clinical course (17, 18). In the above mentioned analysis, the patients' median age was 8 years with a predominance of males (55.4%) (16). Similarly, our patient was a 9-year-old boy. Obesity was acknowledged as the most common underlying disorder in the cohort of children with PIMS mentioned above, being noticed in 30.5% of Hispanic patients, 27.5% of black and only 6.6% of white children (16). Nevertheless, our patient was a white Caucasian obese patient.

In terms of symptoms, it was already reported that gastrointestinal symptoms are more common in children than adults (19). The first cluster of children with this severe multisystem inflammatory syndrome temporally associated to COVID-19 was initially reported in England and their symptoms occurred ~2–4 weeks after acute COVID-19, all of them being detected with serological proof of SARS-CoV-2 infection (20). Most-likely, our patient also developed the symptoms ~4 weeks after the infection taking into account his previous contact with the positive mother. The most frequent symptoms identified in this cluster of 78 cases consisted of fever, conjunctivitis, rash, peripheral edema, gastrointestinal symptoms, increased inflammatory markers, cardiac impairment and shock (20). Similar symptoms were detected in the cohort from USA with most of the patients complaining of gastrointestinal symptoms (90.9%), cardiovascular impairment (86.5%), and dermatologic or mucocutaneous involvement (70.9%) (16). In terms of gastrointestinal symptoms, abdominal pain was the most common (61.9%) followed by vomiting (61.8%), and diarrhea (53.2%) (16). Conjunctival injection and skin rash were also frequently encountered in these U.S. children (16). Similar to the previous reports, our patient presented fever, abdominal pain, conjunctival injection and palpebral edema. Viral sepsis caused by SARS-CoV-2 infection is a well-documented condition in adults, and the patients usually experience pulmonary, renal, hepatic, hematologic, and cardiac involvement, often with positive RT-PCR from respiratory swabs (21). Moreover, a recent study performed on Latin American children proved that in terms of lung impairment, ground-glass opacities and consolidations caused by this virus are commonly associated to severe forms requiring intensive care admission, and even death (22). Similarly, bacterial sepsis displays an increased risk for multiple organ failure in children (23). Contrariwise, in children with PIMS there is a notable absence of pulmonary and renal failure, usually with D-dimers as the single modified coagulation parameter (20), differences that might explain the relatively favorable evolution despite their admission in the pediatric intensive care unit since it was pointed out that more than 50% of the children diagnosed with PIMS usually require mechanical ventilation or inotropic support (16, 20). Nevertheless, our patient did not require admission in the pediatric intensive care unit since he did not experience cardiac or respiratory dysfunctions. It is well-documented that the symptoms of Kawasaki disease usually overlap with the presenting symptoms of PIMS, but it rarely leads to a need for admission in a pediatric intensive care unit (24, 25). Moreover, a large retrospective study pointed out that elevated D-dimers at the time of admission represent a reliable predictor of bleeding and thrombotic events, as well as subsequent critical illness and death (26). The authors indicated as well a significant association between acute phase markers, both CRP and ESR, and thrombosis (26). Therefore, albeit controversial, anticoagulation therapy might benefit the patients with thrombotic risk after a thorough assessment. A comparative analysis between the most common causes of hyperinflammatory syndrome in children revealed that anemia is a common laboratory finding in patients with Kawasaki disease, but not PIMS associated to COVID-19 (27). Moreover, anemia was not commonly reported either in children with acute COVID-19 (28, 29). Thus, we hypothesize that in our patient anemia was most likely caused by the interrelation between obesity and PIMS since the single presence of either of them was not previously reported to cause anemia.

As we already mentioned above, the diagnosis of PIMS requires at least one of the following evidences of SARS-CoV-2 infection: positive serology, antigen or RT-PCR (12–14). Although the association between positive serology and RT-PCR is commonly encountered in patients with this condition, out of the 565 U.S. children, 46.1% has only positive serology, while 25.8% tested positive only at RT-PCR (16). In our patients we found both positive serology and RT-PCR for SARS-CoV-2. The treatment of PIMS usually requires a multidisciplinary approach with close monitoring and intensive care support. Previous reports mentioned that the most commonly used therapies in children with this severe condition include intravenous immunoglobulin, steroids, antiplatelet medication, anticoagulation therapy, and other supportive therapies, i.e., respiratory or inotropic support (16). Both European Society for Emergency Medicine and European Academy of Pediatrics stated that optimal supportive care is the mainstay of treatment in children with PIMS since there are no evidence-based recommendations to support other efficacious targeted therapies for this condition (30). Taking into account the highly resemblance between PIMS and Kawasaki disease, the authors recommend the same management as in the latter condition involving intravenous immunoglobulins, steroids, biological therapies, and aspirin associated with early cardiovascular support in the setting of toxic shock (30). Moreover, intravenous steroids were proven to be highly effective in children with Kawasaki disease preventing the development of long-term complications like coronary aneurisms (31, 32). Based on these findings and in the lack of other general consensus, intravenous immunoglobulin and steroids seem to be a reliable choice in children with PIMS associated to COVID-19. Moreover, the early diagnosis and proper treatment are time crucial for these patients since it was proved that they usually respond well to treatment with a full and quick recovery if no delays in treatment occur. Our patient also responded well to the treatment, with no side-effects during the treatment, presenting a full recovery within ~14 days.

Taking into account all the above-mentioned facts and the long-term life-threatening risks that emerge in pediatric patients with obesity, among which type 2 diabetes mellitus, non-alcoholic fatty liver disease, dyslipidemia, and hypertension that were proved to occur even in small ages in the setting of obesity, it is imperiously necessary to be aware of the additional risks of the actual viral pandemics and their short-term life-threatening complications in these patients (33). Additionally, recent longitudinal studies indicated that adolescents with severe obesity carry a great risk for cardiomyopathy, heart failure, and cardiovascular mortality during adulthood increasing their mortality rates (33). Other studies revealed even an association between obesity and increased risk for pancreatic cancer, hepatocellular carcinoma, breast tumors, and colon cancer (34). Thus, it is incontestable that childhood obesity, a current global burden, with alarmingly increasing incidence predisposes children to develop severe forms of COVID-19, the emerging pandemics. A close monitoring of all children with obesity and COVID-19 is mandatory in order to preempt further complications, as well as a thorough follow-up for ~6 weeks after the acute phase of COVID-19 in order to favor an early diagnosis of PIMS associated to this condition. Thus, all laboratory parameters should be assessed periodically in these patients taking into account that the interrelation between obesity and COVID-19 might result in life-threatening complications, intensive care unit admission and invalidating sequelae in the lack of a proper multidisciplinary approach and complex treatment.

## Conclusions

The pediatricians worldwide are facing two current eras that seem to be intricated, one displays the alarming incidence of pediatric obesity and the second consists of the challenging COVID-19 pandemic. It was proved that children with obesity express a systemic inflammatory involved in a wide-spectrum of well-documented life-threatening complications, which seems to be a potential risk factor for PIMS-associated to COVID-19. Thus, a timely diagnosis of PIMS in children with obesity is mandatory for the best outcome of these patients. Clinicians should be aware that every child with fever lasting more than 5 days could develop this severe hyperinflammatory syndrome. Children with obesity and COVID-19 should be considered a peculiar patient group and benefit from a proper long-term follow-up of ~6 weeks in order to preempt the development of PIMS since serious sequelae might occur if this condition is not recognized and treated in a timely manner.

## Data Availability Statement

The raw data supporting the conclusions of this article will be made available by the authors, without undue reservation.

## Author Contributions

COM, LEM, and MOS conceptualized and designed the study, drafted the initial manuscript, and reviewed and revised the manuscript. MOS and LEM designed the data collection instruments, collected data, carried out the initial analyses, and reviewed and revised the manuscript. All authors approved the final manuscript as submitted and agree to be accountable for all aspects of the work.

## Conflict of Interest

The authors declare that the research was conducted in the absence of any commercial or financial relationships that could be construed as a potential conflict of interest.

## Abbreviations

ALT: alanine aminotransferase
AST: aspartate aminotransferase
COVID-19: Coronavirus disease 2019
ESR: erythrocyte sedimentation rate
PIMS: Pediatric inflammatory multisystem syndrome
RT-PCR: real-time polymerase chain reaction
SARS-CoV-2: severe acute respiratory syndrome-2
WHO: World Health Organization.
